# Potency Determination of Antidandruff Shampoos in Nystatin International Unit Equivalents

**DOI:** 10.4103/0250-474X.45413

**Published:** 2008

**Authors:** D. B. G. Anusha Hewage, W. Pathirana, Amara Pinnawela

**Affiliations:** Department of Pharmacology and Pharmacy, Faculty of Medicine, University of Colombo, Kynsey Road, Colombo-08, Sri Lanka; 1 Microbiology Department, National Drugs Quality Assurance Laboratory, No.120 Norris Canal Road, Colombo-10, Sri Lanka

**Keywords:** Antidandruff Shampoo, nystatin, Nystatin international unit equivalents (NIUEqs)

## Abstract

A convenient standard microbiological potency determination test for the antidandruff shampoos was developed by adopting the pharmacopoeial microbiological assay procedure of the drug nystatin. A standard curve was drawn consisting of the inhibition zone diameters vs. logarithm of nystatin concentrations in international units using the fungus *Saccharomyces cerevisiae* (yeast) strain National Collection of Type Culture (NCTC) 1071606 as the test organism. From the standard curve the yeast inhibitory potencies of the shampoos in nystatin international unit equivalents were determined from the respective inhibition zones of the test samples of the shampoos. Under test conditions four shampoo samples showed remarkable fungal inhibitory potencies of 10227, 10731, 12396 and 18211 nystatin international unit equivalents/ml while two shampoo samples had extremely feeble inhibitory potencies 4.07 and 4.37 nystatin international unit equivalents/ml although the latter two products claimed antifungal activity. The potency determination method could be applied to any antidandruff shampoo with any one or a combination of active ingredients.

Shampoos may be classified into three distinct categories. Plain detergent shampoos that are more like soaps in their properties and uses, the cosmetic shampoos with conditioners, dyes or other cosmetic ingredients used for cosmetic purposes and the medicated shampoos, which are formulated to contain any drug substance as in the case of antidandruff shampoos. Each such shampoo category must be dealt accordingly for regulatory purposes. Medicated antidandruff shampoos containing typical drugs such as climbazole^1^ as the active ingredient are qualifying them to be classified and evaluated for regulatory purposes as drugs. These shampoos are however grouped under cosmetics. The shampoo trade is becoming very diverse. Many of these products claim to be effective against the fungal infection of the scalp associated with dandruff. The infective fungi belong to the genus *Malassezia* also known as *Pityrosporum*, which is a tiny yeast^2^. The medicated shampoos evade being subjected to routine quality control since they are not treated as drugs and also probably for want of a convenient standard analytical procedure applicable to the whole range of antifungal shampoos with diverse active ingredients.

The antifungal drug nystatin consists of a mixture of biologically active substances consisting of tetraene molecules A_1_, A_2_ and A_3_^3^,^4^ and is microbiologically assayed according to the pharmacopoeias^4^,^5^ using *Saccharomyces cerevisiae* as the test organism. It was thought that this procedure could be adopted to screen the shampoos claiming antidandruff activity since a fungus is also responsible for infections associated with dandruff. The proposed microbiological assay method is further justified as both nystatin and the antifungal shampoos are aimed at treating superficial fungal infections^6^ and also because both nystatin and the antidandruff shampoos may also consist of a mixture of substances making chemical analysis very complex. The potency of a given shampoo is expressed in terms of nystatin international unit equivalents per milliliter abbreviated NIUEqs and pronounced nukes. Five brands of antifungal shampoos claiming to contain as the active ingredient one of either zinc pyrithione, climbazole or selenium sulfide and a sixth brand with an antidandruff claim but without any declared antifungal agent were subjected to fungal inhibitory activity determination by the proposed method. A standard curve was drawn using four geometric dilutions of nystatin as permitted in the British Pharmacopoeia^5^. Prior to carrying out the main assay dilutions of the shampoo samples whose inhibition zone diameters corresponded with that of the inhibition zone region represented in the standard curve were determined.

Six brands of shampoos claiming antidandruff activity were procured from the market. Three of them contained zinc pyrithione^7^ 1.0%, one contained climbazole^8^ without indicating the strength, another had selenium sulfide^1^ 2.5% and the sixth product did not indicate any active ingredient although it had claimed antifungal activity.

Nystatin standard serial dilutions containing 200, 100, 50 and 25 international units per milliliter were prepared in pH 6 buffer containing 5% dimethylformamide as per the British Pharmacopoeia. *Saccharomyces cerevisiae* strain NCTC 1071606 was used as the inoculating agent in the Medium F as per the pharmacopoeia. The inoculum was adjusted to contain 10^6^ organisms per milliliter by diluting the yeast suspension with sterile physiological saline. This adjustment was determined spectrophotometrically and corresponds to an absorbance value 0.5 when measured at 630 nm. The cell suspension was stored at 4° until use. Sterile procedures were carried out in Yamato Model ADS-160 clean bench. A standard curve consisting of log nystatin concentration in international units vs inhibition zone diameters was drawn based on the data in the Table 1.

**TABLE 1:** DILUTIONS AND THE CORRESPONDING INHIBITION ZONES FOR THE NYSTATIN STANDARD CURVE

Nystatin dilutions (I.U./ml)	Log nystatin dilutions (I.U./ml)	Mean inhibition zone diameters (mm±SD)
200	2.3010	33.0±0.32
100	2.0000	30.6±0.13
50	1.6989	27.4±0.26
25	1.3979	24.5±0.16

IU: International Units of nystatin. SD: Standard deviation of the mean diameters (n = 9).

The inhibition zones for standard nystatin solutions were determined by incubating the inoculated petri dishes containing 100 μl nystatin standard dilutions per well at 30° for 18 h. Centre well of each Petri dish was filled with 100 μl of the blank buffer solution as the control. For each dilution inhibition zones were determined in triplicate and three diameters for each inhibition zone were recorded. Prior to the main analysis serial dilutions of the shampoo samples were prepared aseptically with the pH 6 buffer containing 5% dimethylformamide and the dilutions corresponding to inhibition zones falling within the range of the standard curve were determined. Four shampoo samples had to be diluted 250 times and two were tested without diluting. Inhibition zones for each shampoo sample were also determined in triplicate using 100 μl per well and three diameters for each inhibition zone were recorded. The centre well was filled with 100 μl of the blank buffer solution as the control. The main potency determination assay was carried out simultaneously for the nystatin standard curve and the appropriately diluted shampoo samples using the same culture media lot under the same conditions.

Since the antidandruff shampoos have diverse active ingredients it is difficult to test each one of them requiring different analytical methods. The study was designed to evaluate any of the shampoos with respect to an equivalent in terms of a reference material. Nystatin was selected as the reference antifungal agent since a microbiological antifungal assay for this substance is prescribed in the British Pharmacopoeia. For a given shampoo with any one of the antifungal agents the fungal inhibitory potency will be indicated in terms of Nystatin International Unit Equivalents per milliliter (NIUEqs/ml). In other words it may be assumed that that much nystatin as shown by the number of NUIEqs/ml was found in the particular shampoo in place of the actual antifungal agent facilitating easy comparison of their potencies.

NIUEqs value of a shampoo is determined as follows. Find out the log nystatin concentration from the standard curve based on Table 1 data relevant to the mean diameter of the inhibition zones of the shampoo in question. Convert this value into antilog. Multiply the antilog value by dilution factor of the shampoo which gives the final potency of the antidandruff shampoo in NIUEqs/ml. Inhibition zones of the shampoo samples and their NIUEqs are given in Table 2.

**TABLE 2:** MEAN INHIBITION ZONE DIAMETERS AND THE CORRESPONDING NIUEqs OF THE SHAMPOO SAMPLES

Antidandruff shampoos	Mean inhibition zone diameters. (mm±SD)	Log nystatin concentration from the standard curve data. (IU/ml)	Nystatin concentration. (IU/ml)	Dilution factor.	NIUEqs/ml
Brand-A.	29.0±0.89	1.8624	72.8450	250	18211.25
Brand-B.	26.8±0.40	1.63272	42.9259	250	10731.47
Brand-C.	26.5±0.54	1.61184	40.9109	250	10227.72
Brand-D.	27.5±0.54	1.69536	49.5861	250	12396.52
Brand-E.	17.3±0.51	0.64092	4.3744	None	4.3744
Brand-F.	17.0±0.70	0.6096	4.0700	None	4.0700

SD: Standard deviation of the mean diameter (n = 9). IU: International units of nystatin. NIUEqs: Nystatin International Unit Equivalents of the shampoo. Brand-A. ‘Head and Shoulders’ by Proctor and Gamble, Thailand: Zinc Pyrithione 1.0%. Brand-B. ‘Pantene pro-v Antidandruff’ by Proctor and Gamble, Thailand: Zinc Pyrithione 1.0%. Brand-C. ‘Sunsilk antidandruff’ by Uniliver, Sri Lanka: Zinc Pyrithione, strength not mentioned. Brand-D. ‘Lifebouy antidandruff’ by Uniliver, Sri Lanka: Active ingredient not mentioned in the label. Brand-E. ‘Dandex Plus’ by Hemas Manufacturing, Sri Lanka: Climbazole, strength not mentioned. Brand-F. ‘Selsun’ by Abbott, Karachi: Selenium sulphide 2.5% w/v.

The outcome of the assay shows that most antidandruff shampoos are effective fungal inhibitors as claimed but there are others with spurious claims. As expected linearity of the nystatin standard curve fell within the narrow range of log transferred nystatin concentrations of 50 to 200 I.U./ml. Four shampoos had inhibition zones corresponding to the standard curve region after diluting 250 times while two others had barely visible zones when tested undiluted. Under the experimental conditions the six shampoos had highly variable potencies 4.07, 4.37, 10227, 10731, 12396 and 18211 NIUEqs/ml in the brands F, E, C, B, D and A, respectively. It may be noted that the potency of nystatin is expressed in International Units (IU) and that of a shampoo expressed in NIUEqs. An obvious difference exists between the *In vitro* experimental conditions and the actual usage of the shampoos. Further studies are required to match the antifungal potencies determined here with that of the antifungal effects under actual conditions of usage.

A comparatively simple microbiological potency determination method using NIUEqs based on a pharmacopoeial assay procedure has given successful results. The newly proposed antidandruff shampoo potency parameter NIUEqs harmonize the potency determination irrespective of the diversity of the antidandruff agents found in the shampoos. The method is suitable for the screening of diverse antifungal agents that may be found in the shampoos. Zinc pyrithione appear to be the most effective antifungal agent among the samples screened. Shampoos with false antidandruff action claims may be easily detected by the proposed method.
